# P-1035. Assessment of Healthcare Providers' Knowledge and Decision-Making Patterns in Peripheral Venous Catheter Management in Acute Care Settings

**DOI:** 10.1093/ofid/ofaf695.1230

**Published:** 2026-01-11

**Authors:** Heather Young, Carolyn Valdez, Diana Mancini, Sarah Gardiner, Timothy C Jenkins

**Affiliations:** Denver Health, Denver, CO; Denver Health, Denver, CO; Denver Health, Denver, CO; Denver Health, Denver, CO; Denver Health, Denver, CO

## Abstract

**Background:**

Peripheral intravenous catheters (PIVC) are a common medical intervention and source of complications for hospitalized patients.

This study assessed awareness of PIVC presence and characterized who makes decisions about PIVC. We hypothesize that RNs are most aware of PIVC presence and are regarded as most appropriate to make decisions about PIVC need.

**Table 1.**
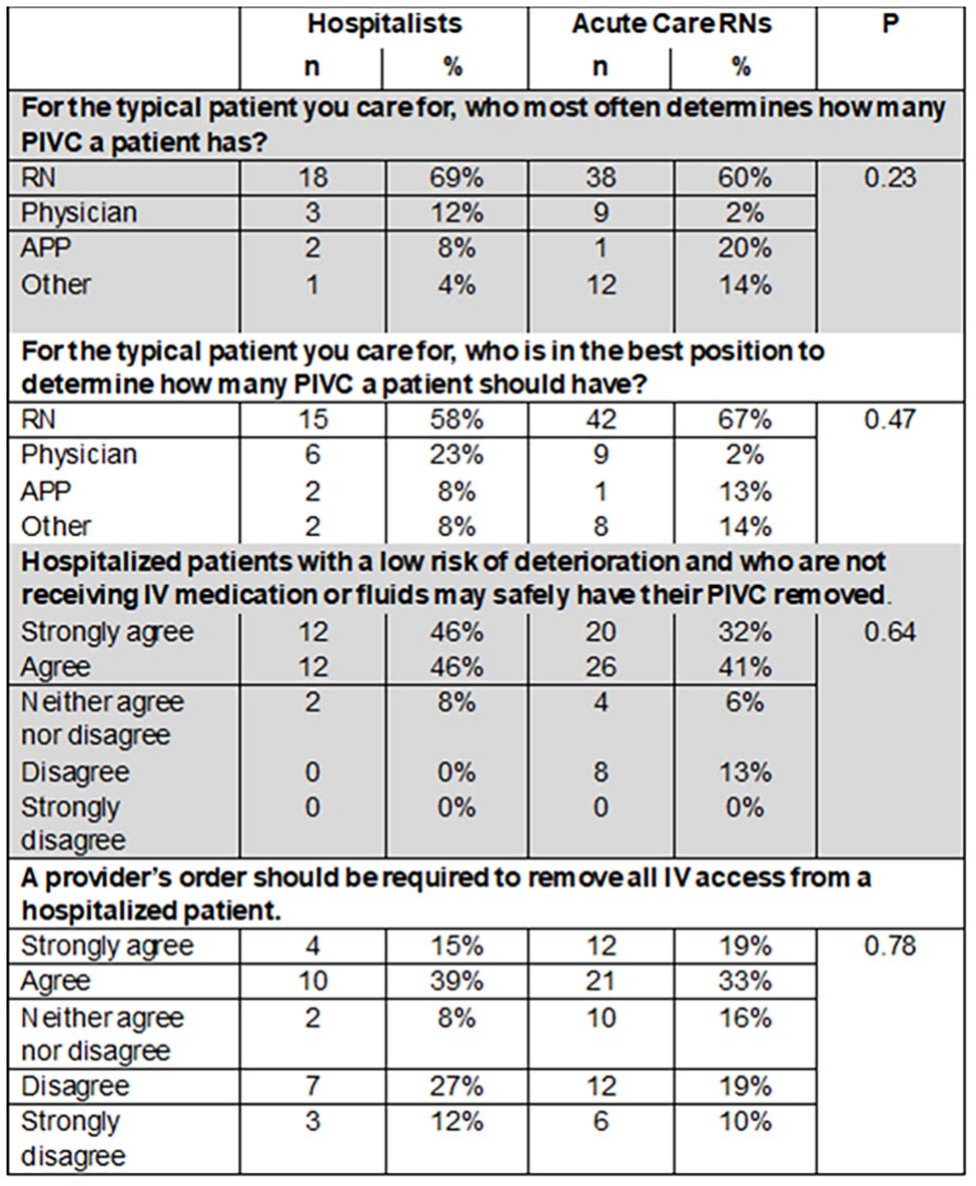
Select survey responses

**Methods:**

This is a cross-sectional, anonymous survey of hospitalists and acute care RNs working on adult units at a 500-bed academic safety net hospital in Denver, CO. The survey was administered between 10/30/24 and 11/30/24.

**Results:**

The response rate was 36% (26/56 hospitalists; 63/193 acute care RNs).

*PIVC awareness.* RNs were more likely to agree or strongly agree that they were aware of how many PIVC their patients had than hospitalists (100% vs 46%, p< 0.00001).

*PIVC decisions* (Table 1).

- Both hospitalists and RNs reported that RNs most often decide the quantity of PIVC
- RNs were identified as being in the best position to decide how many PIVC a patient needed
- Most hospitalists and RNs agreed or strongly agreed that IV access can be safely discontinued for hospitalized patients with a low risk of deterioration who are not receiving IV medications or fluids
- Over half of respondents agreed or strongly agreed that a provider’s order is necessary to discontinue all IV

**Conclusion:**

Despite RNs having the greatest awareness of PIVC and being regarded as best to make the decisions about PIVC, less than half of respondents felt that RNs should remove PIVC without an order.

**Disclosures:**

All Authors: No reported disclosures

